# *Helicobacter pylori* infection and antioxidants can modulate the genotoxic effects of heterocyclic amines in gastric mucosa cells

**DOI:** 10.1007/s11033-013-2622-3

**Published:** 2013-05-10

**Authors:** Tomasz Poplawski, Cezary Chojnacki, Anna Czubatka, Grazyna Klupinska, Jan Chojnacki, Janusz Blasiak

**Affiliations:** 1 Department of Gastroenterology and Internal Diseases, Medical University, Lodz, Poland; 2 Department of Molecular Genetics, University of Lodz, Lodz, Poland

**Keywords:** *Helicobacter pylori*, DNA damage, DNA repair, PhIP, MeIQx, IQ, Melatonin, Vitamin C, Heterocyclic amines

## Abstract

*Helicobacter pylori* (*H. pylori*) infection plays an important role in gastric carcinogenesis. This bacterium may induce cancer transformation and change the susceptibility of gastric mucosa cells to various exogenous dietary irritants. The aim of the study was to evaluate the influence of *H. pylori* infection on the reaction of the stomach cells to a genotoxic effect of heterocyclic amines (HCAs). These well-known mutagens are formed during cooking of protein-rich foods, primarily meat. Taking into account that persons consuming a mixed-western diet are exposed to these compound nearly an entire lifetime and more than half of human population is infected with *H. pylori*, it is important to assess the combined effect of *H. pylori* infection and HCAs in the context of DNA damage in gastric mucosa cells, which is a prerequisite to cancer transformation. We employed 2-amino-3-methylimidazo[4,5-f]quinoline (IQ), 2-amino-3,8-dimethyl-imidazo[4,5-f]quinoxaline (MeIQx) and 2-amino-1-methyl-6-phenylimidazo[4,5-b]pyridine (PhIP) because these substances are present in a great amount in cooked and fried meat. Using alkaline comet assay, we showed that the extent of the DNA damage induced by HCAs was significantly higher in *H. pylori* infected gastric mucosa cells than in non-infected counterparts. We did not observed any difference in the efficiency of repair of DNA lesions induced by HCAs in both type of cells. Vitamin C reduced the genotoxic effects of HCAs in *H. pylori* infected and non-infected gastric mucosa cells. Melatonin more effectively decreased DNA damage caused by HCAs in *H. pylori* infected gastric mucosa cells as compared with control. Our results suggest that *H. pylori* infection may influence the susceptibility of gastric mucosa cells to HCAs and dietary antioxidative substances, including vitamin C and melatonin may inhibit the genotoxic effects of HCAs on gastric mucosa cells and may reduce the risk of carcinogenesis caused by food borne mutagens and *H. pylori* infection.

## Introduction

*Helicobacter pylori* (*H. pylori*) is a gram-negative, spiral-shaped bacterium that colonize the stomach in more than half of the world population. This bacterium has been classified as a definite carcinogen for gastric cancer by the International Center for Cancer Research on the basis of numerous epidemiological studies [1]. The mechanism through which *H. pylori* exerts its carcinogenic effect is primarily associated with the expression of the CagA protein. It was reported that CagA-negative *H. pylori* strains could also initiate cancer transformation, but the mechanisms of carcinogenesis in this case is associated with chronic gastritis. In this inflammatory condition the immune cells try to kill bacterial cells and synthesize large amounts of free radicals and aldehydes and gastric cells are exposed to these radicals, gastric juice, *N*-nitroso compounds, which are thought to play a secondary, after CagA, role in the development of gastric cancer associated with *H. pylori* infection [2].

*Helicobacter pylori* could also initiate cancerogenesis indirectly by disturbing normal gastric cells metabolism and in consequence sensitize them to dietary mutagens. We showed previously that *H. pylori* infection can modulate the susceptibility of gastric mucosa cells to *N*-methyl-*N*’-nitro-*N*-nitrosoguanidine (MNNG), considered as a dietary mutagen and vitamin C and melatonin reduced the genotoxic effect of MNNG [3]. These antioxidant substances are able to scavenge free radicals and prevent the formation of nitrosamines. However in individuals *H. pylori* infected have been observed reduced levels of antioxidant compounds, such as vitamin C and increased ROS activity. This could be main reason for stomach diseases and gastric carcinoma [4]. Another group of mutagens presented in food are heterocyclic amines (HCAs). HCAs are formed by food processing. This group of chemicals is of a special significance, because HCAs may directly interact with DNA and form mutagenic adducts [5]. HCAs are present mainly in high temperature-processed meat and fish [6]. They arise as a product of protein pyrolysis or the Maillard reaction. To date 22 HCAs have been found in the pyrolysates of amino acids and proteins, they were divided depending on their chemical structure into two groups, IQ type and non-IQ type. IQ type includes aminoimidazoles, such as 2-amino-3-methylimidazo[4,5-f]-quinoline (IQ), 2-amino-3,8-dimethylimidazo [4,5-f]quinoxaline (MeIQx) and 2-amino-1-methyl-6-phenylimidazo[4,5-b]pyridine (PhIP), and non-IQ type includes pyridoindoles and dipyridoimidazoles, such as 3-amino-1,4-dimethyl- 5H-pyrido[4,3-b]indole (Trp-P-1) and 2-amino-6- methyldipyrido[1,2-a:3′,2′-d] imidazole (Glu-P-1) [5, 6]. HCAs of IQ type are abundant in thermally prepared meats. The degradation of HCAs in humans is initiated by the reactions of *N*-oxidation, which are catalyzed mainly by the cytochrome P-450 enzymes [7]. The next step involves an enzymatic esterification by acetyltransferases or sulfotransferases to produce reactive nitrenium ions, which can form covalent bonds with DNA mainly at the N2 and C8 positions of guanine, and, when not repaired, cause mutations. DNA adducts can be also formed by HCAs without any enzymatic activation [8]. After the metabolic activation of HCA’s, their metabolites are capable of oxidative DNA damage. HCA’s can also induced DNA strand breaks. According to their mutagenic nature it is important to assess the combined effect of *H. pylori* infection and HCAs in the context of DNA damage in gastric mucosa cells, which is a prerequisite to cancer transformation. In the present work we evaluated the influence of *H. pylori* infection on the susceptibility of gastric mucosa cells to the DNA-damaging effect of HCAs. We chose IQ, PhIP and MelQx as representative HCAs, because they are present in a relatively high amount in cooked and fried meat and fish [9, 10]. In addition, we checked the protective potential of vitamin C and melatonin against the genotoxic action of HCAs in gastric mucosa cells. We also assessed the ability of infected and non-infected GMCs to repair DNA damage, so in general, the aim of the study was to evaluate the influence of *H. pylori* infection on the reaction of the stomach cells to a genotoxic effect of HCAs.

## Materials and methods

### Chemicals

HCAs, IQ (2-amino-3-methylimidazo[4,5-f]quinoline), MeIQx (2-amino-3,8-dimethyl-imidazo[4,5-f]quinoxaline) and PhIP (2-amino-1-methyl-6-phenylimidazo[4,5-b]pyridine were purchased from Toronto Research Chemicals (Toronto, Canada) and dissolved in dimethyl sulfoxide (DMSO, Sigma Aldrich, St. Louis, USA). Proteinase K, collagenase, low-melting and normal-melting point agarose, phosphate buffered saline (PBS), Hanks’ balanced salt solution (HBSS), Tris and EDTA were purchased from Sigma Chemicals (St. Louis, MO, USA). All remaining chemicals were of the highest commercial grade available.

### Patients

Forty patients of Department of Gastroenterology and Internal Diseases, Medical University of Lodz, Lodz, Poland were studied. They underwent endoscopic examination due to gastric complains, they reported. All patients suffered from dyspeptic symptoms—mostly epigastric pains before and between meals. We excluded subjects with organic changes, deep grade of gastritis, any organic disease, neuropsychiatric disorders, a past history of surgical treatment, another functional disease of alimentary tract, particularly irritable bowel syndrome, allergy, food intolerance, non-steroidal and inflammatory drugs therapy. To avoid any potential influence of confounding factors we also excluded subjects with smoking and abuse alcohol history. There was no difference between gender in both groups. 17 of them, aged from 18 to 63, median 44 years, were *H. pylori* infected as confirmed by ^13^C-urea breath test. A value of over 3 ‰ in this test was considered to indicate *H. pylori* infection. Remaining 23 patients (25–65 years, median 47 years) were controls. The study was approved by the Ethics Committee of the Medical University of Lodz and each subject gave a written consent.

### Cell preparation

Human gastric mucosa cells were isolated from tissue sample obtained during upper digestive endoscopy from the greater curvature of the upper corpus and the antrum of the stomach of each individual. Tissue samples were immediately transported to the laboratory in an ice cold HBSS. The samples were then incubated for 1 h at 37 °C in HBSS supplemented with 3 mg/ml of proteinase K and collagenase. The mixture was gently mixed by pipetting every 15 min. The gastric mucosa cells were harvested by centrifugation for 15 min at 150×*g* and washed twice with PBS. The final suspension of the cells was adjusted to 10^6^ per ml with HBSS and further processed.

### Cell treatment

HCAs were added from their 1 M DMSO solution to the cell suspension to give final concentrations 0.05–0.15 mM. The control cells received only the growth medium with the chemical vehicles supplementation (DMSO and/or methanol). The final concentrations of DMSO and methanol were adjusted to the identical level in all samples. To examine DNA damage cells were incubated with HCAs for 1 h at 37 °C. Each experiment included a positive control, which was hydrogen peroxide at 10 μM for 15 min on ice, producing a pronounced DNA damage in the gastric mucosa cells. To assess the effect of vitamin C and melatonin on DNA damage, cells were preincubated with vitamin C at 50 μM or melatonin at 10 μM for 30 min and incubated with IQ, PhIP and MelQx at 50 μM. Vitamin C was taken from a stock solution in water (20 mM). Melatonin was derived from a stock (10 mM) solution in methanol. The chemicals vehicles, DMSO and methanol, at the concentrations presented in the samples, did not affect the processes under study (data not shown).

### Comet assay

The comet assay was performed under alkaline conditions essentially according to the procedure of Singh et al. [11] with modifications [12] as described previously [13].

### Data analysis

The values in this study were expressed as mean ± SEM from three experiments, i.e. the data from three experiments were pooled and the statistical parameters were calculated. Prior to statistical analysis we have used a normality test (Kolmogorov–Smirnov) to check that the two populations follow a normal distribution. The Mann–Whitney test was used to determine differences between samples with distribution departing from the normality. The differences between samples with the normal distribution were evaluated by the Student’s *t* test. Data analysis was performed using SigmaStat software (v. 3.0.0, SPSS, Chicago, USA).

## Results

### *Helicobacter pylori*-infected gastric mucosa cells display a higher level of basal endogenous DNA damage than their non-infected counterparts

**Fig. 1:**
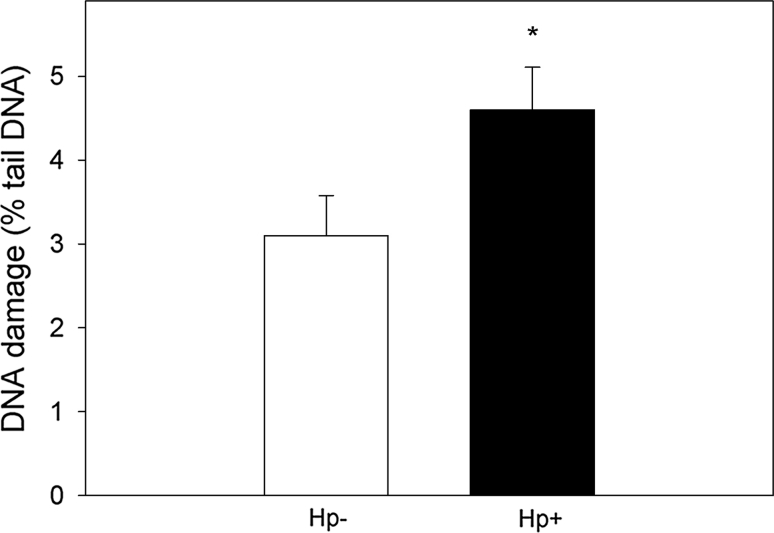
Basal endogenous DNA damage measured as the mean comet tail DNA in alkaline comet assay of *H. pylori*-infected (*white column*) and non-infected (*black column*) gastric mucosa cells. 17 individuals were analyzed in the infected group and 23 in the non-infected (control) group. The number of cells scored for each individual was 50 and the analysis was repeated three times. *Error bars* denote SEM, *p < 0.05, as compared with the non-infected cells

The mean basal endogenous DNA damage detected by the alkaline comet assay, measured as % tail DNA in gastric mucosa cells of *H. pylori*-infected and non-infected patients is displayed in Fig. 1. The extent of DNA damage in *H. pylori*-infected patients was greater than in the non-infected ones (*p* < 0.05).

### IQ, PhIP and MeIQx induce DNA damage

**Fig. 2:**
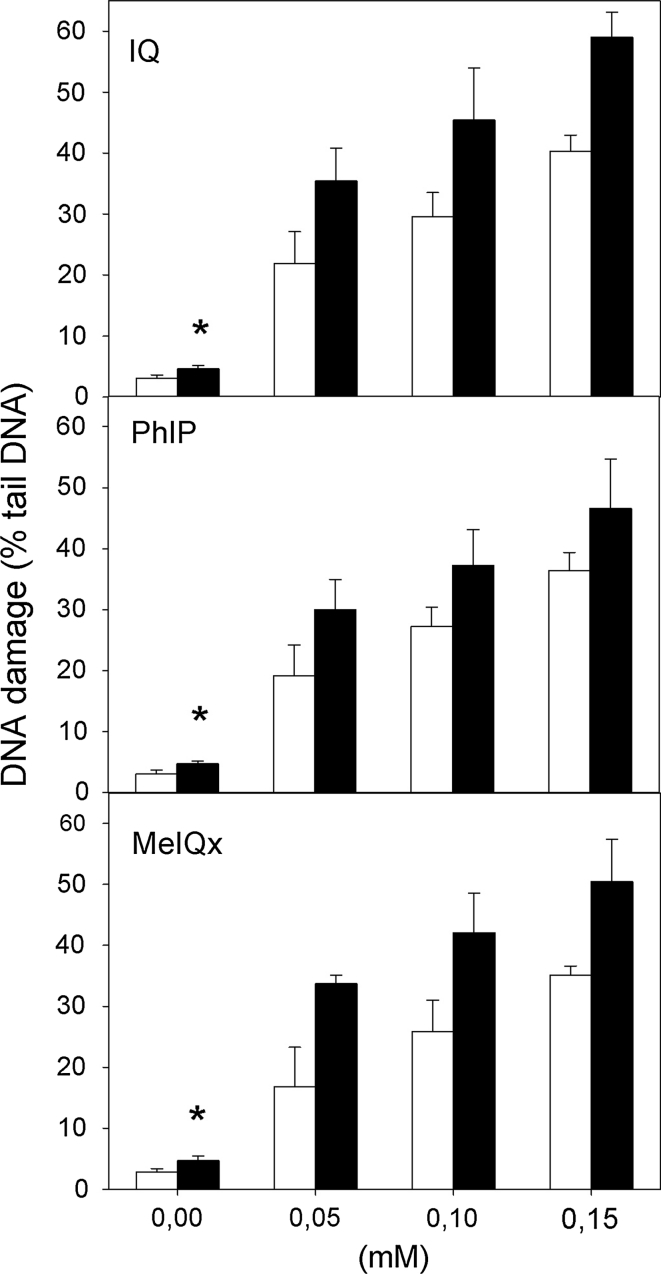
DNA damage measured as the percentage of DNA in the tail of a comet in the alkaline comet assay of *H. pylori*-infected (*black column*) and non-infected (*white column*) gastric mucosa cells exposed for 1 h at 37 °C to 2-amino-3-methylimidazo[4,5-f]quinoline (IQ), 2-amino-1-methyl-6-phenylimidazo[4,5-b]pyridine (PhIP) and 2-amino-3,8-dimethyl-imidazo 4,5-f]quinoxaline (MeIQx). 17 individuals were analyzed in the infected group and 23 in the non-infected (control) group. The number of cells scored for each individual was 50 and the analysis was repeated three times. *Error bars* denote SEM,*p < 0,001

Figure 2 shows DNA damage measured in the alkaline version of the comet assay in *H. pylori* infected and non-infected human gastric mucosa cells exposed for 1 h to IQ, PhIP and MeIQx at 50, 100 and 150 μM. Single and double DNA strand breaks as well as alkali labile sites can be detected in this version of the technique. All HCAs induce a concentration dependent increase in the percentage of DNA in the comet tail (*p* < 0.001 for each concentration). The extent of DNA damage was higher in *H. pylori* infected than in non-infected gastric mucosa cells (*p* < 0.05).

### Vitamin C and melatonin protect gastric mucosa cells against the DNA-damaging effect of HCAs

**Fig. 3:**
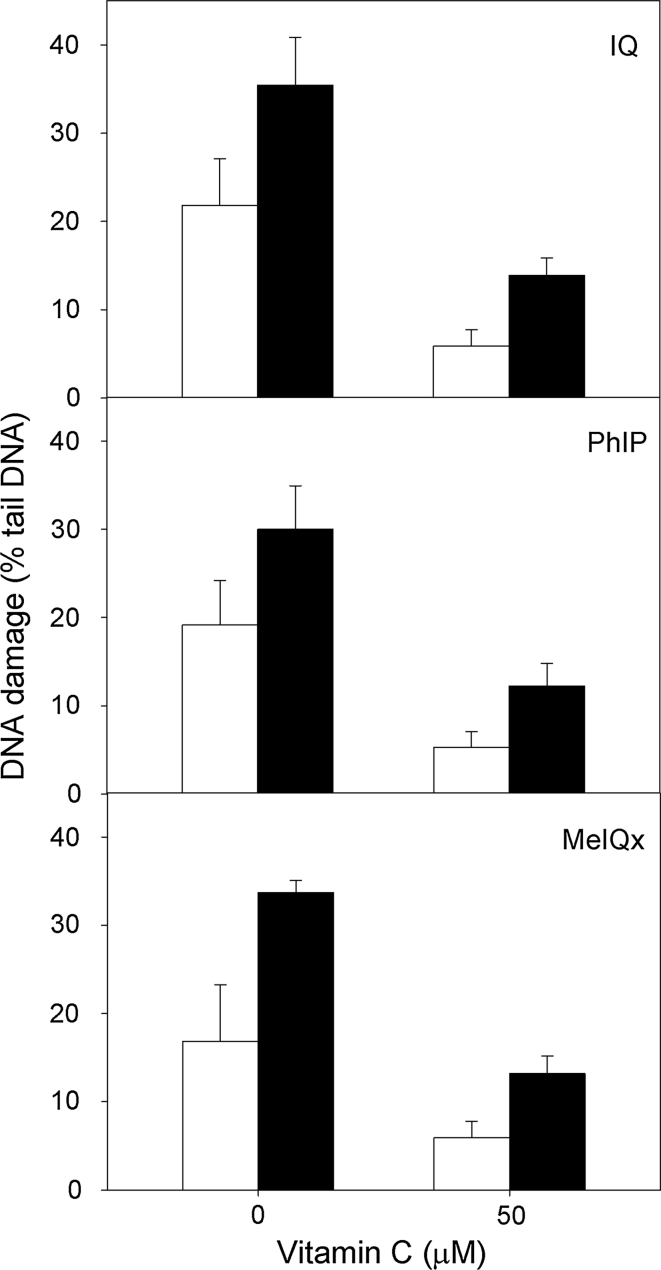
DNA damage measured as the percentage of DNA in the tail of a comet in the alkaline comet assay of *H. pylori*-infected (*black column*) and non-infected (*white column*) human gastric mucosa cells exposed for 1 h at 37 °C to 2-amino-3-methylimidazo[4,5-f]quinoline (IQ), 2-amino-1-methyl-6-phenylimidazo[4,5-b]pyridine (PhIP) and 2-amino-3,8-dimethyl-imidazo 4,5-f]quinoxaline (MeIQx) after preincubation with vitamin C at 50 μM. 17 individuals were analyzed in the infected group and 23 in the non-infected (control) group. The number of cells scored for each individual was 50 and the analysis was repeated three times. *Errors bars* denote SEM, *p < 0.01

**Fig. 4:**
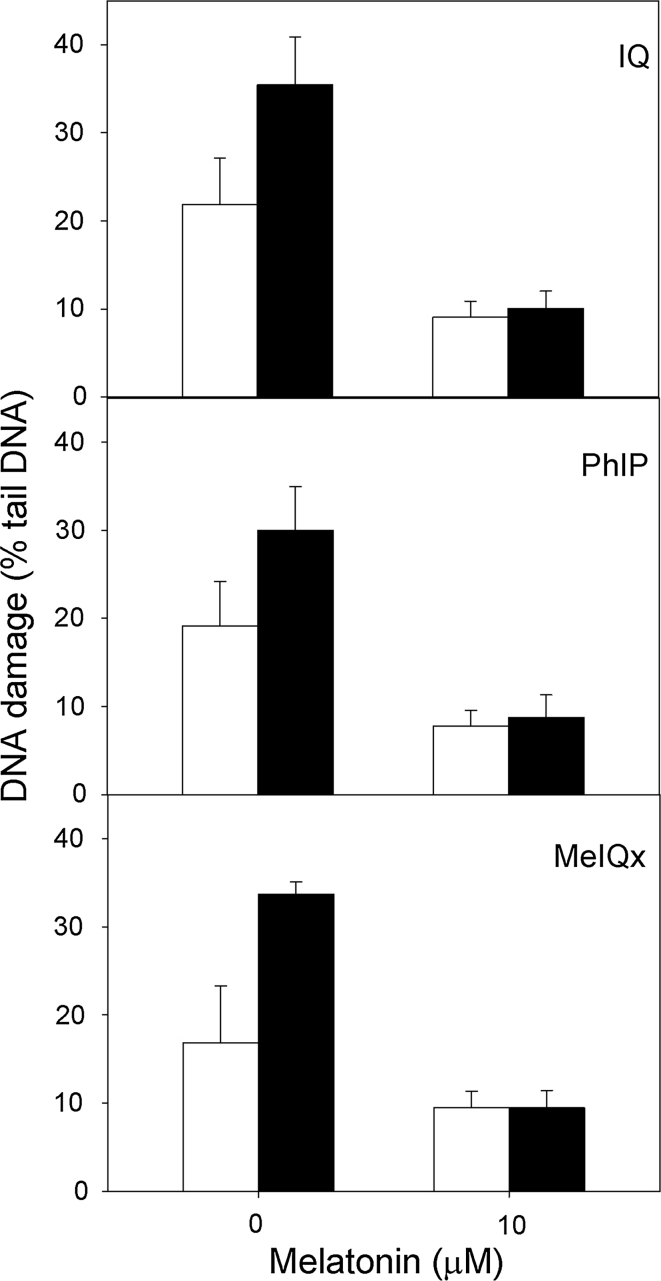
DNA damage measured as the percentage of DNA in the tail of a comet in the alkaline comet assay of *H. pylori*-infected (*black column*) and non-infected (*white column*) human gastric mucosa cells exposed for 1 h at 37 °C to heterocyclic amines: 2-amino-3-methylimidazo[4,5-f]quinoline (IQ), 2-amino-1-methyl-6-phenylimidazo[4,5-b]pyridine (PhIP) and 2-amino-3,8-dimethyl-imidazo 4,5-f]quinoxaline (MeIQx) after preincubation with melatonin at 10 μM. 40 individuals were analyzed in the infected group and 23 in the non-infected (control) group. The number of cells scored for each individual was 50 and the analysis was repeated three times. *Errors bars* denote SEM, *p < 0.05

Both vitamin C and melatonin exerted a protective effect; the effect was more pronounced in the infected cells (relative ratio of DNA damage without and with vitamin C in infected cells—2.32, in non-infected—4.11, *p* < 0.01; Fig. 3). They also showed that the repair of the DNA damage induced by HCAs is faster in the infected cells (3.10 versus 2.53, *p* < 0.05, Fig. 4).

### *Helicobacter pylori*-infected gastric mucosa cells display a faster initial kinetics of the repair of DNA damage induced by HCAs than their non-infected counterparts

**Fig. 5:**
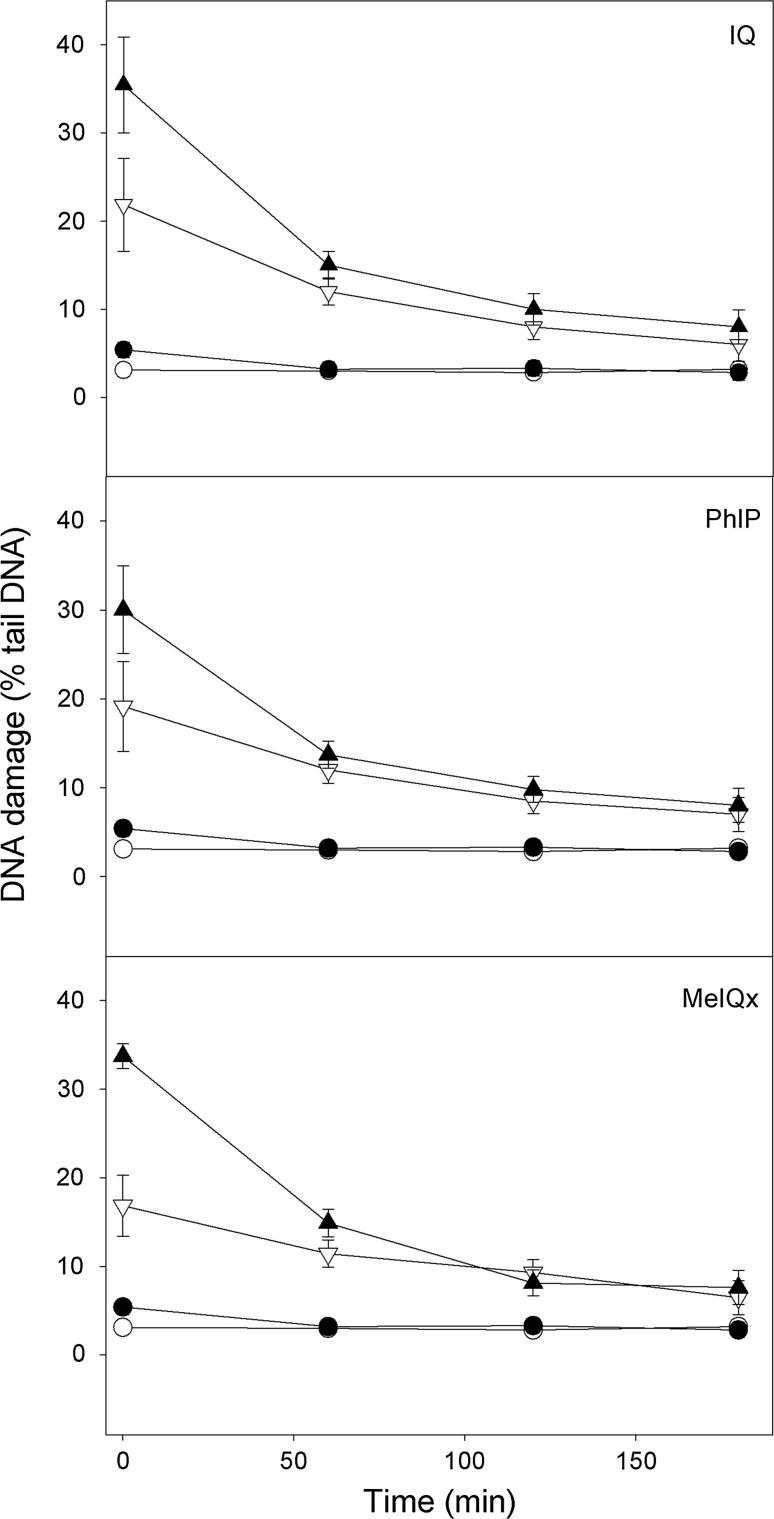
The time course of the repair of DNA damage measured as the percentage of DNA in the tail of comets in the alkaline comet assay in *Helicobacter pylori*-infected (*black*) and non-infected (*white*) gastric mucosa cells treated (*triangles*) for 1 h at 37 °C to heterocyclic amines: 2-amino-3-methylimidazo[4,5-f]quinoline (IQ, A), 2-amino-1-methyl-6-phenylimidazo[4,5-b]pyridine (PhIP, B) and 2-amino-3,8-dimethyl-imidazo 4,5-f]quinoxaline (MeIQx, C). Cells were treated with a chemical, washed and incubated in a repair, heterocyclic amine-free, medium for 3 h at 37 °C. Untreated cells (*dots*) served as controls. 40 individuals were analyzed in the infected group and 23 in the non-infected (control) group. The number of cells scored for each individual was 50 and the analysis was repeated three times. *Error bars* denote SEM

We analyzed the kinetics of DNA repair in gastric mucosa cells after HCAs treatment by measuring the extent of DNA damage in the cells exposed to either HCA at 50 μM immediately after the exposure as well as 60, 120 and 180 min thereafter (Fig. 5). The comet tail DNA of the control cells was constant, indicating that preparation and subsequent processing of the cells did not introduce a significant damage to their DNA. The cells exposed to 10 μM hydrogen peroxide (positive control) were able to recover within 60 min (results not shown). The cells exposed to IQ, PHiP and meIQx at 50 μM were able to remove more than 90 % of the damage to their DNA within 180 min (*p* < 0.001). The character of the kinetics of DNA repair was similar in the infected as non-infected gastric mucosa cells, but the former displayed a faster kinetics during approximately the first 60 min because at this time-point, the extent of DNA damage in both kinds of cells did not differ significantly (*p* > 0.05) and remained the same till the end of the repair incubation.

## Discussion

*Helicobacter pylori* infection induce the generation of intracellular ROS in gastric epithelial cells [14]. During this infection the host organism switches on an immune response associated with a considerable amount of free radicals and other harmful compounds. Independently of this response, some strains of the bacterium may produce the CagA protein, which may affect the stability of the gastric mucosa cells. Both phenomena may contribute to cancer transformation in the gastric mucosa. In the present work we wanted to assess, whether *H. pylori* infection may modulate the response of gastric mucosa cells to dietary mutagens, HCAs, and whether the infection affect also the action of known free radicals scavengers, vitamin C and melatonin. We have chosen 1 h incubations of gastric cells with HCAs, because food did not lie heavy on the stomach and the exposition time on food derived mutagens is relative short as compared with colon. Our results indicate that infection of *H. pylori* might increase the susceptibility of human gastric cells to HCAs. If we think about the mechanism of the interaction of a chemical with cellular DNA, firstly we should ask a question about the permeability of the plasma membrane to this chemical. In fact, urease, an enzyme of *H. pylori,* produces ammonia, which may destabilize the integrity of gastric mucosa cells membrane by the activation of neutrophils production, accelerated by free radicals produced by *H. pylori* and host immune cells [15]. Another mechanism of the changes in the permeability of the gastric mucosa cells membrane is associated with vacuolating cytotoxin (VacA) protein produced by *H. pylori*. VacA can cause a wide range of alterations in eukaryotic cells in vitro. VacA has the ability to induce the formation of large cytoplasmic vacuoles and permeabilization of the plasma membrane [16, 17], reduction of the mitochondrial transmembrane potential and release of cytochrome c from the mitochondrium [18, 19], inhibition of the activation and proliferation of T lymphocytes [20], and activation of mitogen-activated protein kinases [21]. Many cellular effects of VacA are attributed to the insertion of VacA into the membrane and the formation of membrane channels [22]. VacA was also reported to reduce the level of cellular glutathione (GSH) [23]. As a major route for the detoxification of HCAs metabolites *per se* is by their reaction with GSH. *H. pylori* decreases the ratio of HCAs utilization in gastric cells and consequently increase the time of action of reactive HCAs metabolites [24].

The main route of metabolic activation of HCAs includes oxidation to hydroxylamines, primarily by CYP1A2 but also by other CYP isoforms. HCAs are also substrates for NAT2 enzyme [25]. Expression of these enzymes was observed in gastrointestinal tract tissues including stomach, so stomach cells metabolize HCAs [26, 27]. There was no data suggesting the role of *H. pylori* (if any) on deregulations of cellular enzymes that metobolize HCAs. Our result might suggest that enhanced toxicity of HCAs in *H. pylori* infected gastric mucosa cells in comparision to non-infected cells was caused by higher rate of metabolic activation of HCAs in presence of *H. pylori*, but this hypothesis needs further studies. These studies should include CYP isoforms and NAT2 enzymes expression profile analysis in *H. pylori*-infected and non-infected gastric cells and phenotype genotype correlation study with most common clinical associated SNP polymorphism of CYP and NAT2 enzymes. Our results show a significantly higher susceptibility of *H. pylori*-infected than non-infected gastric mucosa cells to HCAs, which is in general agreement with those reported by our group previously and Ladeira et al. [3, 28, 29]. On the other hand Everett et al. [30] showed that *H. pylori* infection decreased the extent of DNA damage observed in gastric mucosa cells. This is not in contrary to our results, since we investigated the induced extent of DNA damage. Moreover, we showed, that the in the first 60 min the infected cells repair their DNA with a faster kinetics than non-infected.

Our results show that DNA lesions induced by HCAs may be reduced by melatonin and vitamin C. Antioxidants levels used in this study correspond to concentrations observed in the gastric juice [31, 32]. Our previous studies have shown that use higher concentrations of this substances lead to DNA damage that is visible in comet assay [3]. Melatonin is a scavenger of a number of reactive oxygen and nitrogen species, and has an ability to reduce lipid peroxidation and may be used as a preventive antioxidant/free radicals scavenger [33]. Moreover, melatonin display some favorable effects in the gastrointestinal tract and can be used as an auxiliary drug in the treatment of some diseases of the tract [34]. The molecular mechanism of a protective action of melatonin in our experiment may be, at least in part, explained by its anti-inflammatory properties. Because melatonin may increase the concentration of GSH in the cell and regulate the activity of several enzymes involved in the detoxification process, it is therefore possible that it may eliminate the GSH-reducing effect of VacA in gastric cells [35, 36]. Our data showing a better protective effect against DNA lesions induced by HCAs in *H. pylori* infected than non-infected cells support this hypothesis.

We observed that vitamin C decreased the extent of DNA lesions induced by HCAs in a *H. pylori* infection-dependent manner. Our data are in general agreement with those showing that vitamin C decreased mutation frequency induced by HCAs in rats [37]. It seems that the vitamin C may influence in some extent the metabolism of HCAs in rodents cells, because the results of other study performed on *Salmonella typhimurium* did not confirm the protective effect of vitamin C on DNA damage induced by HCAs.

HCAs are mutagens that are formed during the cooking of protein-rich foods such as meat or fish. Since these products are common in the diet worldwide, it is important to decrease their potential harmful consequences. Our results suggest that vitamin C and melatonin can be beneficial in this respect, but people infected with *H. pylori* should avoid taking food with HCAs, because the infection may potentiate the genotoxic action of these compounds, and such an action may sponsor cancer transformation of the gastric mucosa cells.
